# Efficacy and safety of 3CL protease inhibitors in patients with mild or moderate COVID-19: a systematic review and meta-analysis of randomized controlled trials

**DOI:** 10.1186/s12985-025-02899-0

**Published:** 2025-08-21

**Authors:** Nicole Jean Lee, Eric Shih Katsuyama, Christian Ken Fukunaga, Wilson Falco Neto, Ana Carolina Covre Coan, Hilaria Saugo Faria, Eduardo Alexandrino Medeiros

**Affiliations:** 1 Faculty of Medicine, Federal University of São Paulo, R. Botucatu, 740, São Paulo, Brazil; 2 Department of Medicine, FMABC University Centre, São Paulo, Brazil; 3 Faculty of Medicine, UNIFIPA University Centre, Sao Paulo, Brazil; 4 Faculty of Medicine, Federal University of Espirito Santo, Vitoria, Espirito Santo Brazil; 5 Faculty of Medicine, Federal University of Santa Maria, Santa Maria, Rio Grande do Sul Brazil; 6 Department of Infectious Diseases, Federal University of São Paulo (UNIFESP), Sao Paulo, Brazil

**Keywords:** COVID19, 3CL protease inhibitors, Mild to moderate, Meta-analysis

## Abstract

**Background:**

Remdesivir has been established as a safe treatment for patients with COVID-19. However, given the SARS-CoV-2 random mutations, 3CL protease inhibitors have been studied in recent randomized controlled trials. Therefore, this meta-analysis aims to compare 3CL protease inhibitors versus placebo in patients with mild to moderate COVID-19.

**Methods:**

We systematically searched PubMed, Embase, and Cochrane Central for RCTs comparing the treatment regimens in patients with mild to moderate COVID-19. Outcomes of interest were the number of patients with symptomatic resolution, recovery rates, viral load change from baseline, alleviation rates, any adverse events, and serious/severe adverse events. Risk ratios (RRs) and hazard ratios (HRs) with 95% CI were calculated for binary outcomes, and standardized mean differences (SMDs) were calculated for continuous outcomes. R version 4.3.2 was used for statistical analysis with the random effects model.

**Results:**

Ten studies were included, comprising 8,511 patients, of whom 4,654 (55.97%) received 3CL protease inhibitors. Follow-up ranged from 21 to 29 months. In our time-to-event analysis, 3CL protease inhibitors significantly increased the rate of alleviation (HR 1.17; 95% CI [1.09;1.25]; *p* < 0.01), and recovery rates (HR 1.18; 95% CI [1.11;1.26]; *p* < 0.01) in patients with mild to moderate COVID-19. However, 3CL protease inhibitors significantly reduced viral load at 72 h (SMD − 0.95; 95% CI [-1.23; -0.66]; *p* < 0.01) compared with placebo in these patients. There was no significant difference between groups in symptomatic resolution (RR 1.01; 95% CI [1.00;1.03]; *p* = 0.147).

**Conclusion:**

3CL protease inhibitors significantly reduced the change in the viral load and increased the rate of symptom alleviation and recovery, underscoring their importance as a potential therapeutic option.

**Supplementary Information:**

The online version contains supplementary material available at 10.1186/s12985-025-02899-0.

## Introduction

Severe acute respiratory syndrome coronavirus 2 (SARS-CoV-2) and its variants have caused COVID-19, a highly infectious disease that has led to a pandemic starting in 2019 and lasting over three years, resulting in more than 6 million deaths worldwide [1, 2]. Remdesivir has been established as a safe treatment for patients with COVID-19 [3]. Additionally, ritonavir-boosted Nirmatrelvir, Molnupiravir, and high-titer COVID-19 convalescent plasma have emergency authorizations for treating this disease [2, 3]. Given the random mutations of SARS-CoV-2 and the limitations of existing vaccines, guidelines are frequently updated to incorporate newly emerging therapies that demonstrate greater efficacy [4].

Whether therapies will remain effective against SARS-CoV-2 mutations remains an open question [5]. The 3-chymotrypsin-like (3-CL) protease of SARS-CoV-2 cleaves the viral polyproteins pp1a and pp1ab to produce functional viral proteins and stabilize substrate-binding pockets [6, 7]. As an important target for small-molecule therapies, it may play an important role against newly emerging variants [8]. Consequently, SARS-CoV-2 3CL protease inhibitors have emerged as promising therapeutic candidates for the treatment of patients with mild to moderate COVID-19, given their potential effectiveness against both current and future variants [7]. However, these individual trials had a small sample size and statistical power, which limits generalizability of their findings [9–11].

Despite the availability of antiviral agents and monoclonal antibodies, the emergence of new SARS-CoV-2 variants with potential resistance mechanisms has underscored the need for broadly effective antiviral strategies. Notably, agents such as Nirmatrelvir, Simnotrelvir, Olgotrelvir, Ensitrelvir, and Leritrelvir are examples of 3CL protease inhibitors that have been evaluated in clinical trials designed to assess their safety and efficacy in patients with COVID-19 [7, 10, 12–16]. Therefore, we aimed to perform a systematic review with meta-analysis of randomized controlled trials (RCTs) comparing the efficacy and safety of SARS-CoV-2 3CL protease inhibitors with placebo in patients with mild and moderate COVID-19, to elucidate their therapeutic role.

### Methods

This systematic review and meta-analysis were performed and reported according to the Preferred Reporting Items for Systematic Reviews and Meta-Analysis (PRISMA) Statement guidelines and the Cochrane Collaboration Handbook for Systematic Reviews of Interventions guidelines [17, 18]. The prospective meta-analysis protocol has been uploaded to the International Prospective Register of Systematic Reviews (PROSPERO; CRD42024562860).

### Eligibility criteria

Inclusion in this meta-analysis was limited to studies that met all the following eligibility criteria: RCTs; enrolling patients with COVID-19 with mild or moderate symptoms; comparing 3CL protease inhibitors with placebo; reporting at least one of the outcomes of interest, and COVID-19 diagnosis requiring confirmation by RT-PCR or other approved molecular testing methods. Mild-to-moderate COVID-19 was defined according to the original criteria of each study. In general, mild cases included symptomatic patients without respiratory compromise, while moderate cases involved evidence of lower respiratory tract involvement (e.g., cough, dyspnea, or radiographic abnormalities) with oxygen saturation (SpO₂) > 93% on room air and no need for supplemental oxygen [19]. The 3CL protease inhibitors considered included RAY1216, Simnotrelvir, Nirmatrelvir, GST-HG171, Ensitrelvir, and Olgotrelvir. There were no restrictions on language or publication date. Observational studies, studies with overlapping patient populations, without a control group, head-to-head comparisons between 3CL protease inhibitors, abstracts, editorials, letters to the editor, preprints, reviews, systematic reviews, and meta-analyses were excluded.

### Search strategy and study selection

We conducted a systematic review of PubMed, Embase, and Cochrane Library on April 1st, 2024. The search strategy was adapted to the different databases according to support for special characters. References of eligible papers, previous systematic reviews, and meta-analyses were also searched for additional studies of interest.

Two reviewers (C.F and W.N) conducted the search, imported results into Rayyan software, and triaged the studies. After excluding duplicates and titles/abstracts unrelated to the clinical question, we assessed the eligibility of each remaining study based on a full-text review of the articles. In instances of disagreement, we consulted a third reviewer (N.L). A full description of our search strategy can be found in Supplementary Methods 3.

### Data extraction

Two independent authors (C.K e W.N) extracted the data in a double-blinded method; in case of any conflict, a third author was consulted. The following data from individual studies was extracted: (1) country; (2) blinding method; (3) number of patients; (4) type of 3CL protease inhibitor; (5) dosage; (6) follow-up in days; (7) mean age; (8) sex distribution; (9) vaccination status proportion; (10) proportion of patients with risk factors for severe COVID-19; (11) proportion of patients with Covid-19 symptoms at baseline; (12) proportion of patients with stratified genetic variants; and (13) initial virus copy number. A full disclosure on how the Kaplan-Meier Curve extraction was performed can be found in our Supplemental Methods 4.

### Endpoints and subgroup analysis

We defined our primary endpoints as the number of patients with symptomatic resolution, viral Ribonucleic Acid (RNA) loading change from baseline, and any adverse events. We collected data on the following secondary endpoints: recovery rate, sustained alleviation rate, and severe/serious adverse events.

We performed a prespecified subgroup analysis of low-dosage (125 mg) versus high-dosage (250 mg) on patients undergoing Ensitrelvir for the viral load endpoint. A full definition of the outcomes can be found in Supplementary Methods 5. Additionally, a post-hoc subgroup analysis was conducted between the different 3CL-inhibitors drugs for the symptomatic resolution endpoint.

### Risk of bias and evidence quality assessment

Six authors (N.L., E.K., C.F., W.N., A.C., and H.F.) conducted the risk of bias assessment in pairs with a double-blind model. Risk of bias in selected randomized trials was assessed using the second version of the Cochrane Risk of Bias assessment tool (RoB 2) [20], evaluating five domains for each outcome of the selected studies: (I) bias in the randomization process; (II) bias due to deviations from intended interventions; (III) bias due to missing data; (IV) bias in outcome measurement; and (V) bias in the selection of the reported results.

The overall risk of bias assessment for each trial outcome was derived from individual domain judgments. After discussing the reasons for the discrepancy, disagreements were resolved through consensus. We also performed funnel plot analysis to appraise publication bias [21].

### Statistical analysis

Statistical analyses were conducted using R (R Foundation for Statistical Computing, Vienna, Austria) version 4.3.2 under the “meta” package [22]. Restricted Estimated Maximum Likelihood (REML) random-effects models were employed for data synthesis. Treatment effects for dichotomous endpoints were compared using Risk ratio (RR) with corresponding 95% confidence intervals (CI), while continuous outcomes were assessed using Standardized Mean Difference (SMD). Time-to-event outcomes, such as recovery and symptom alleviation rates, were calculated using Hazard Ratio (HR) and 95% CIs. Statistical significance was defined as a p-value < 0.05. Heterogeneity was evaluated through I² statistics and Cochran’s Q test, with significance defined as *p* < 0.10 and I² >40%. Sensitivity analyses were conducted to ensure the robustness and reliability of the findings for outcomes with high heterogeneity.

### Trial sequential analysis

We used the TSA 0.9.5.10 Beta software for trial sequential analysis (TSA) [23] to confirm our meta-analysis results. The type of boundary value for the hypothesis test was set to a two-sided test with an alpha value of 5%. Once the cumulative studies in the Z-curve cross the conventional monitoring boundary or the futility area, the results are consistent and should be considered reliable evidence.

## Results

**Fig. 1:**
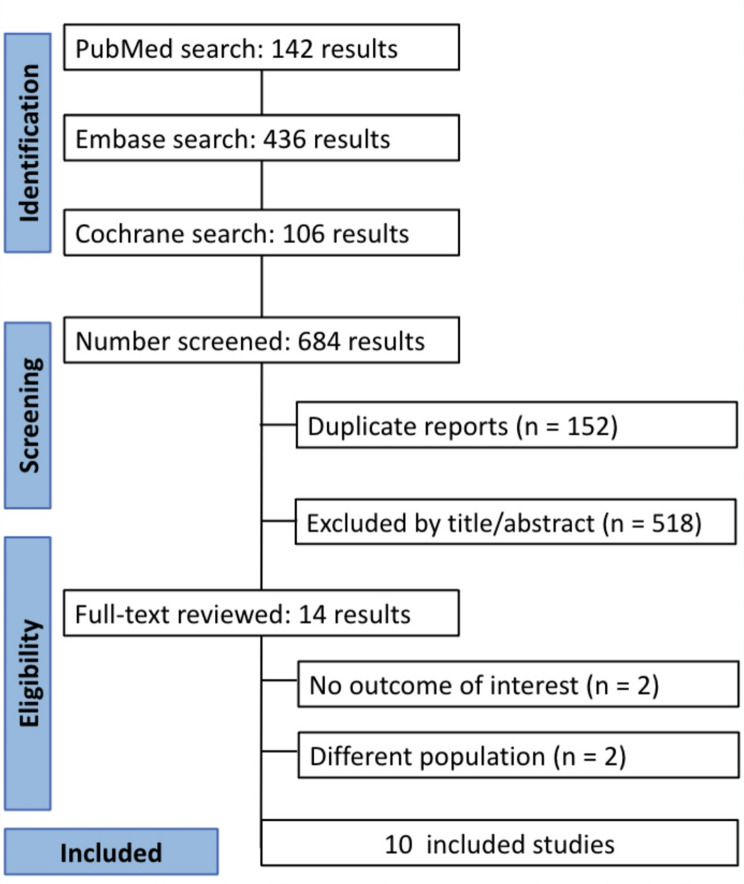
PRISMA flow diagram of study screening and selection

The preliminary search yielded 684 studies from three databases: 142 studies from PubMed, 436 from Embase, and 106 from the Cochrane Library. After title and abstract screening and removal of duplicates, 14 studies were assessed for full-text analysis following the established inclusion and exclusion criteria. A total of 10 RCTs [9–16, 24, 25] were identified, comprising 8,511 patients, of whom 4,654 (55.97%) received Sars-Cov-2 3CL protease inhibitors. (Fig. 1). A full definition of eligibility criteria can be found in Supplementary Results 1.

Baseline characteristics of the included studies are presented in Table 1. All studies were double-blind randomized controlled trials (RCTs), with the majority conducted in China (60.00%). The mean follow-up duration was 27.6 months (range: 21–29 months). The mean age of participants was 37.3 years; 4,148 (48.73%) were male, and 2,039 (23.95%) had risk factors for severe COVID-19. Regarding vaccination status, 7,599 participants (89.28%) had received at least one dose of a COVID-19 vaccine. A detailed definition of the recovery/alleviation time assessed in each study, as well as the COVID-19 symptoms evaluated, is provided in Supplementary Results 1.

### Pooled analysis of all studies

**Fig. 2:**
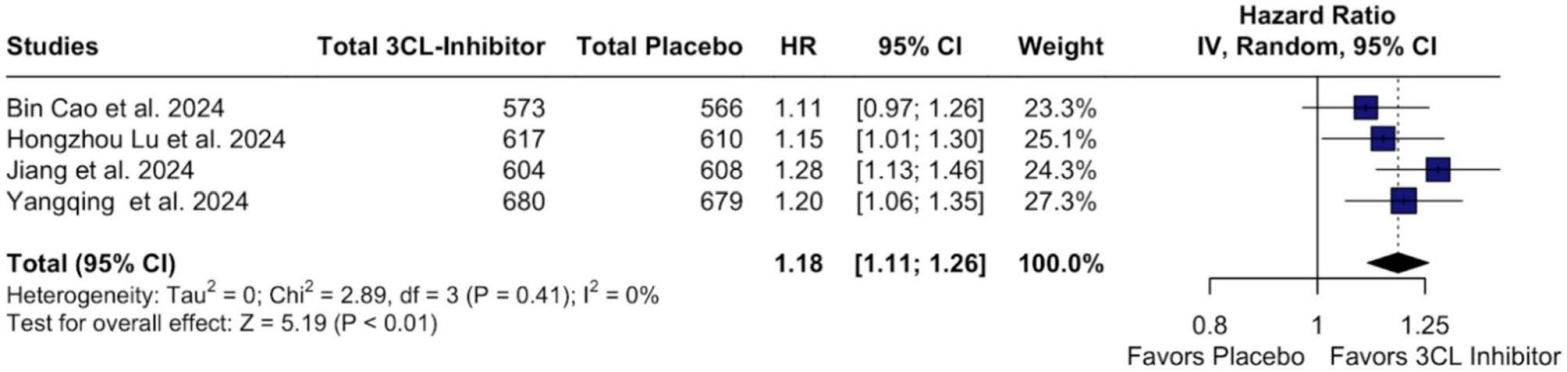
Forest Plot: Time to event for sustained COVID-19 recovery *Legend*: The rate to time-to-event for sustained recovery COVID-19 recovery is 18% higher in patients in 3CL-inhibitor therapy. *Abbreviations*: 3CL:3-chymotrypsin-like; CI: Confidence Interval; HR: Hazard Ratio; IV: inverse variance

**Fig. 3:**
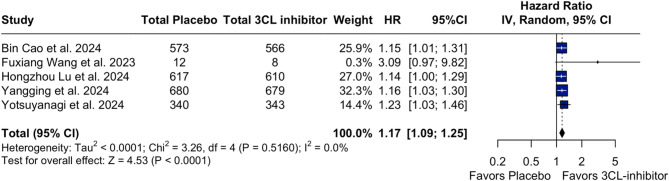
Forest Plot: Time to event for sustained COVID-19 alleviation *Legend*: The risk to time-to-event for sustained alleviation COVID-19 recovery is 17% higher in patients in 3CL-inhibitor therapy. *Abbreviations*: 3CL:3-chymotrypsin-like; CI: Confidence Interval; HR: Hazard Ratio; IV: inverse variance

**Fig. 4:**
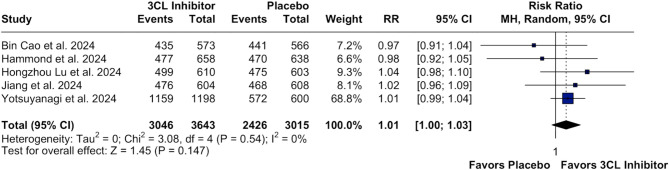
Forest Plot: Number of patients with resolution of COVID-19 *Legend*: There is no difference in the number of resolutions of COVID-19 in patients in 3CL inhibitors therapy compared to placebo. *Abbreviations*: 3CL:3-chymotrypsin-like; CI: Confidence Interval; IV: inverse variance; MH: Mantel-Haenszel; RR: Risk Ratio

**Fig. 5:**
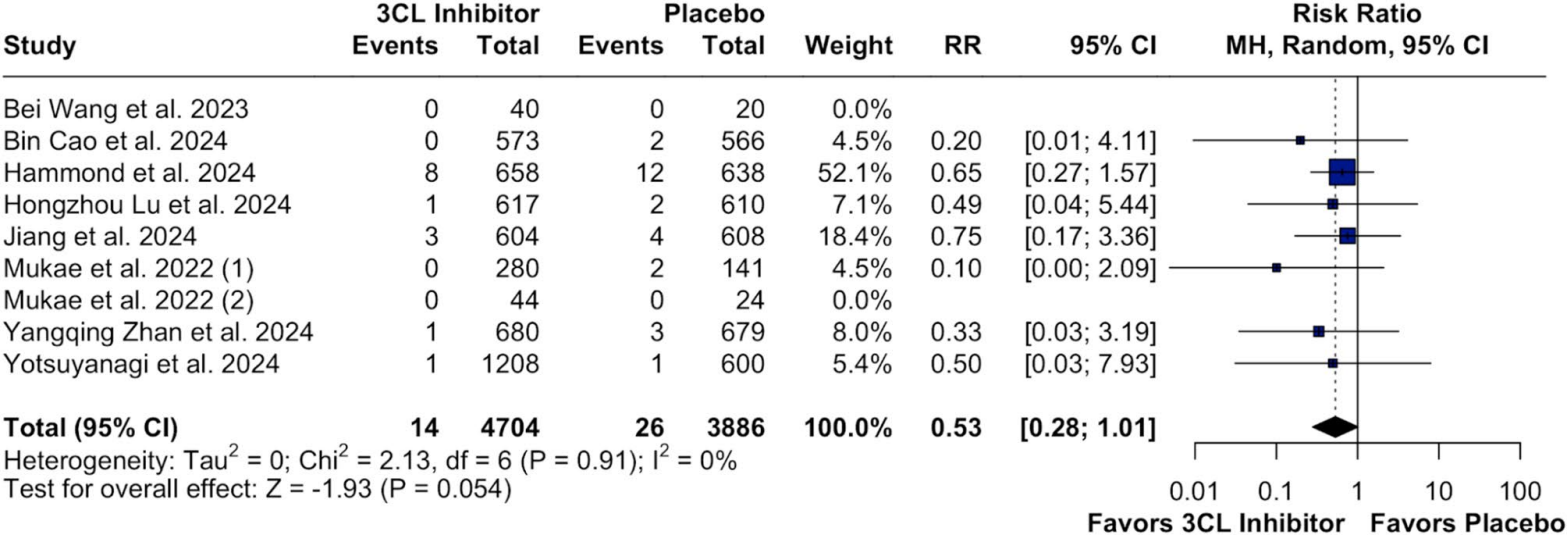
Forest Plot: Severe or Serious Adverse Events *Legend*: There is no difference in risk severe or serious adverse events of COVID-19 in patients in 3CL inhibitors therapy compared to placebo. *Abbreviations*: 3CL:3-chymotrypsin-like; CI: Confidence Interval; IV: inverse variance; MH: Mantel-Haenszel; RR: Risk Ratio

In our time-to-event analysis, 3CL protease inhibitors significantly increased rate of recovery (HR 1.18; 95% CI [1.11;1.26]; p < 0.01; I²=0%; Fig. 2) and alleviation rate (HR 1.17; 95% CI [1.09;1.25]; p < 0.01; I²=0%; Fig. 3) of clinical COVID-19 symptoms at baseline compared with placebo. There was no statistically significant difference between groups in the number of patients with resolution of symptoms (RR 1.01; 95% CI [1.00;1.03]; p = 0.147; I²=0%; Fig. 4) and in terms of the occurrence of serious/severe adverse events (RR 0.53; 95% CI [0.28;1.01]; p = 0.054; I²=0%; Fig. 5). However, there was a significant increase in the 3CL protease inhibitors group regarding the incidence of any adverse event (40.24% vs. 31.26%; RR 1.25; 95% CI [1.06;1.48]; *p* = 0.010; I²=86%; Supplementary Fig. 1) compared with placebo.

Our results showed that 3CL protease inhibitors significantly reduced the viral load change from baseline in 48 h (SMD − 1.15; 95% CI [-1.38;-0.92]; *p* < 0.01; I²=34%; Supplementary Fig. 2A), 72 h (SMD − 0.95; 95% CI [-1.23; -0.66]; *p* < 0.01; I²=76%; Supplementary Fig. 2B), 96 h (SMD − 1.30; 95% CI [-1.80; -0.79]; *p* < 0.01; I²=88%; Supplementary Fig. 2C), and 120 h (SMD − 1.15; 95% CI [-1.38; -0.92]; *p* < 0.01; I²=34%; Supplementary Fig. 2D) compared with placebo.

### Subgroup analysis

In a prespecified subgroup analysis restricted to studies including 687 patients undergoing Ensitrelvir therapy with different dosages, the change from baseline in the viral load in 72 h was statistically significant reduced with Ensitrelvir both at 125 mg (SMD − 1.43; 95% CI:-1.56 to -1.29]; *p* < 0.01; I²=0%; Supplementary Fig. 3) and 250 mg dosages (SMD − 1.38; 95% CI: -1.61 to -1.15]; *p* < 0.01; I²=30%; Supplementary Fig. 3). There were no statistically significant difference between the dosages (*p* = 0.74). Our post-hoc subgroup analysis for the different 3CL-inhibitors drugs did not have a significant interaction for the number of symptomatic resolutions endpoint (Supplementary Fig. 4, *p* = 0.57).

### Trial sequential analysis

The Trial Sequential Analysis (TSA) graphs are presented in Supplementary Fig.5. For the outcome “Number of Resolutions” (Supplementary Fig. 5A), the Z-curve reached the required information size (RIS) for both the number of resolutions and serious or severe adverse events. However, when TSA monitoring boundaries were applied, the Z-curve remained within the futility boundaries, indicating no significant difference between the use of SARS-CoV-2 3CL protease inhibitors and controls. For the outcome “Serious Adverse Events” (Supplementary Fig. 5B), the Z-curve did not reach the RIS of 11,069 patients and did not cross the futility boundaries, suggesting that the current evidence is insufficient to determine a difference between the groups.

### Quality assessment

We conducted a bias assessment according to the Risk of Bias (RoB 2) criteria and have presented the results in Supplementary Fig. 6A. Except for the Jiang et al. study, which presented some concerns due to a moderate risk of bias in measurement of the outcome, the other studies showed a low risk of bias. The funnel plot (Supplementary Fig. 6C), demonstrated a symmetrical distribution, suggesting a no small-study effects bias.

## Discussion

In this systematic review and meta-analysis of 10 studies and 8,511 patients, we compared 3CL protease inhibitors with placebo in patients with mild to moderate COVID-19 with a mean follow-up of 27.6 months. The main findings of our analysis were: (1) the number of patients with resolution of symptoms was not significantly different between groups; (2) recovery and symptom alleviation rates showed a favorable trend in patients receiving 3CL protease inhibitors compared to placebo; (3) 3CL protease inhibitors significantly reduced the viral load from baseline as compared with placebo and (4) were associated with higher risk of any adverse events (40.24% vs. 31.26%).

Current guidelines for COVID-19 treatment recommend nirmatrelvir-boosted ritonavir for patients with mild to moderate symptoms who are at high risk of progressing to severe disease [26]. However, the emergence of new SARS-CoV-2 variants [27] and significant drug–drug interactions associated with nirmatrelvir-ritonavir have limited its use in certain patient populations. As a result, recent studies have investigated novel 3CL protease inhibitors, which may offer broader antiviral activity and address current therapeutic limitations. In this context, our meta-analysis provides timely and relevant evidence on the potential benefits of these agents for both non-hospitalized and hospitalized patients with mild to moderate COVID-19.

In patients with mild to moderate COVID-19, 3CL protease inhibitors did not show an increase in the quantity of patients with symptomatic improvement. In the Bin Cao et al. study [10], 1,208 patients with mild to moderate COVID-19 were randomized to Simnotrelvir or placebo. In the 603 patients who underwent Simnotrelvir plus ritonavir, there was a 1.4% absolute reduction in the number of patients with symptomatic resolution. However, Hongzhou Lu et al. [25], which included 617 patients randomized to GST-HG171, showed an absolute 3.3% decrease in symptomatic improvement in the placebo group. Although these results were not statistically significant, the discrepancy between these studies could be due to the use of different drugs (Simnotrelvir vs. GST-HG171) or the higher number of patients with risk factors in the Bin Cao study (52.7% vs. 11.2%).

This report incorporates a time-to-event analysis for symptom recovery and alleviation to contextualize the findings. Although no significant difference was observed in the absolute number of patients achieving symptom resolution, treatment with 3CL protease inhibitors resulted in a meaningful increase in the rate of recovery and symptom alleviation over time compared to placebo. Notably, each trial assessed a distinct set of COVID-19 symptoms, ranging from 11 to 14 symptoms, except for Wang et al. [11], who evaluated only five. Despite this variability, the pooled analysis demonstrated a statistically significant improvement in both recovery and symptom alleviation. Supporting these findings, an observational study by Han et al. [28] reported a 14% improvement in clinical outcomes among patients with mild to moderate COVID-19 treated with 3CL protease inhibitors compared to placebo, aligning with our results. Compared to other COVID-19 therapies, 3CL-inhibitors presented with similar efficacy results to Molnupiravir [29] while showing greater clinical benefit than Favipiravir [30] and Azithromycin [31].

The measurement of viral load is critical, as it is associated with disease progression and severity [32]. Our meta-analysis indicates a significant reduction from baseline in viral load among patients treated with 3CL protease inhibitors compared to placebo. However, substantial heterogeneity was observed, which widened the 95% confidence intervals and introduced potential ambiguity. This variability likely stems from differences in recruitment criteria, follow-up duration, daily dosages, and baseline patient characteristics. Moreover, disparities in risk factors and symptom severity at enrollment may influence therapeutic response across populations [33].

In the study by Fuxiang Wang et al. [11], patients received either high or low doses of Simnotrelvir plus ritonavir. A significant viral load reduction was observed in both treatment arms compared to placebo, with greater efficacy in the high-dose group [34]. However, this study had a small sample size and included patients with more moderate symptoms and relevant comorbidities. These findings are consistent with the overall results of our meta-analysis. Similarly, a subgroup analysis of patients treated with Ensitrelvir at doses of 125 mg and 250 mg showed a trend toward viral load reduction, though no significant difference was found between the two dosing groups.

Despite the observed improvement in viral load, our analysis also demonstrated a higher incidence of adverse events in the treatment group. These events included any reported health changes during the study period — whether or not they were related to the drug —such as abnormal lab results or mild clinical symptoms. The most commonly reported adverse events were mild and self-limiting laboratory abnormalities, including decreased neutrophil count, diarrhea, nausea, dysgeusia, hypokalemia, and increased triglyceride levels [35]. Elevated serum triglycerides have been previously associated with both Nirmatrelvir [24] and Ensitrelvir [13, 14], and hypokalemia has also been documented in the literature [36]. In the study by Fuxiang et al., three participants developed hypokalemia categorized as treatment-emergent adverse events (TEAEs). Nevertheless, the safety data did not indicate a consistent risk signal attributable to the drug.

Given the prevalence of electrolyte disturbances in COVID-19, particularly hypokalemia, regular monitoring of serum potassium levels is advisable. Importantly, although adverse events were more frequent in the treatment group, they were predominantly mild or moderate. There was no increase in the incidence of severe or serious adverse events compared to placebo. In contrast, the placebo group showed a higher risk for serious adverse events.

This meta-analysis of randomized controlled trials offers robust evidence regarding the efficacy and safety of 3CL protease inhibitors for patients with non-severe COVID-19. Combining data from ten international, multicenter trials—including both hospitalized and non-hospitalized patients with mild to moderate symptoms—provided sufficient statistical power to evaluate multiple outcomes. Notably, while no significant difference in the absolute number of symptom resolutions was observed, the trial sequential analysis (TSA) confirmed that the required information size was reached. Additionally, our time-to-event analyses for symptom recovery and alleviation suggest a benefit for the treatment group over time. Although 3CL protease inhibitors were associated with more frequent adverse events, these were generally mild and did not translate into higher rates of severe adverse outcomes.

Nonetheless, several limitations should be acknowledged. First, there was heterogeneity in the specific 3CL protease inhibitors and dosing regimens used, precluding robust subgroup analyses. Second, the included trials varied in the number and type of COVID-19 symptoms assessed (ranging from 5 to 14), limiting consistency in outcome measurement. Third, viral load analyses exhibited high heterogeneity, as previously discussed. Fourth, missing data for some outcomes restricted the robustness of certain analyses, and individual patient data would enhance the precision of our findings. Lastly, we were unable to perform our prespecified subgroup analysis comparing vaccinated and unvaccinated patients due to insufficient data. Future head-to-head RCTs should address this gap to better understand efficacy in these subpopulations.

## Conclusion

In summary, this systematic review and meta-analysis suggests the potential efficacy and safety of 3CL protease inhibitors in patients with mild to moderate COVID-19. These agents significantly accelerated symptom alleviation and recovery over time compared to placebo. Additionally, they were associated with a greater reduction in viral load from baseline, highlighting their potential as an effective antiviral therapy. While the incidence of adverse events was higher in the treatment group, most events were mild or moderate and not necessarily attributable to the drug. No increase in severe adverse events was observed.

Nonetheless, further randomized controlled trials are warranted to perform detailed subgroup analyses and clarify the drug’s efficacy and safety across different populations, including vaccinated versus unvaccinated individuals.

**Table 1:** Baseline characteristics of included studies

Study and Year	Country	Drug	Dosage, mg	Follow-up, days	No. of patients^+^	Age, y^+^	Male, *n*^+^	Vaccine Status, *n*^+^	Risk factors for severe disease, *n*^+^	Moderate symptoms, *n* ^+^	XBB Genetic variant, *n*^+^	Initial viral load*^+^
**Bei Wang**, 2023 [9]	China	RAY1216	400	21	20/20	39.4/39.9	10/7	20/19	NA	NA	NA	7.9/8.4
		RAY1216 plus RTV¶	300		20/20	37.4/39.9	9/7	20/19				8.0/8.4
**Cao**, 2024 [10]	China	Simnotrelvir plus RTV¶	750	29	573/566	35/35 ^†^	333/340	549/543	302/307	387/347	0/1	6.25/6.36
**Fuxiang Wang**, **2023[11]**	China	SIM0417 plus RTV¶	750	28	12/8	36.3/43.6	11/5	11/8	6/5	1/0	0/0	7.89/7.44
			300		12/8	39.5/43.6	9/5	12/8	4/5	1/0	0/0	7.42/7.44
**Jiang**, 2024 [12]	China	Olgotrelvir	600	29	604/608	34/33‡	212/228	579/588	136/137	36/37	NA	6.2/6.2‡
**Hammond**, 2024 [24]	Mainly USA/Europe	Nirmatrelvir plus RTV¶	300	28	658/638	41/42‡	312/284	374/364	319/317	308/307	NA	5.3/5.0
**Hongzhou Lu**, **2024[25]**	China	GST-HG171 plus RTV¶	150	28	617/610	34.3/34.7	282/256	594/585	69/68	43/52	233/251	6.7/6.9
**Mukae**, 2022(1) [13]^§^	Japan and South Korea	Ensitrelvir fumaric acid	125	28	114/111	35.6/37.3	61/72	97/97	NA	NA	0/0	NA
			250		116/111	35.3/37.3	66/72	97/97			0/0	
**Mukae**, 2022(2) [14]	Japan	Ensitrelvir fumaric acid	125	28	16/17	38.8/38	8/13	14/12	NA	NA	0/0	NA
			250		14/17	40.4/38	8/13	12/12			0/0	
**Yangqing Zhan**, 2024 [15]	China	RAY1216	400	29	680/679	33.7/33.4‡	347/323	672/666	71/75	53/54	2/0	7.5/7.5^†^
**Yotsuyanagi**, 2024 [16]	Japan, Vietnam, South Korea	Ensitrelvir (< 72 h)	125	28	347/343	35.7/34.7	193/174	322/315	107/89	NA	0/0	6.98/6.93
			250		340/343	35.3/34.7	185/174	313/315	90/89		0/0	6.89/6.93
		Ensitrelvir (120 h)	125		603/600	35.9/35.3	318/311	562/553	174/152		0/0	6.83/6.77
			250		595/600	35.9/35.3	323/311	551/553	167/152		0/0	6.73/6.77

Binary data is displayed as a number (%), while continuous data is displayed as mean (standard deviation) unless otherwise specified. ^§^Phase 2 A Study (different recruitment periods); ^*^log10 copies/ml; ^†^median[IQR]; ‡median(range); ^+^Intervention/Placebo; ¶Studies included RTV dosage of 100mg. *Abbreviations*: NA: not available; RTV: ritonavir, RAY1216: Leritrelvir, SIM0417: Simnotrelvir

## Supplementary Information

Below is the link to the electronic supplementary material.

Supplementary Material 1

## Data Availability

All data used for this study has been included in the manuscript and supplementary material.

## Abbreviations

3CL: 3-chymotrypsin-like
CI: Confidence Interval
Fig.: Figure
HRs: Hazard Ratios
MDs: Mean Differences
PRISMA: Preferred Reporting Items for Systematic Reviews and Meta-Analysis
PROSPERO: Prospective Register of Systematic Reviews
RCTs: Randomized controlled trials
REML: Restricted Estimated Maximum Likelihood
RIS: Required Information Size
RNA: Ribonucleic Acid
RRs: Risk ratios
RoB 2: Risk of Bias assessment tool for randomized controlled trials
SARS-CoV-2: Severe acute respiratory syndrome coronavirus 2
SMDs: Standardized Mean Differences
Supp.: Supplemental
TEAE: treatment-emergent adverse events
TSA: Trial Sequential Analysis
