# Supplementary material for: Efficacy and safety of 3CL protease inhibitors in patients with mild or moderate COVID-19: a systematic review and meta-analysis of randomized controlled trials
Source: Virol J. 2025 Aug 21;22:286. doi: 10.1186/s12985-025-02899-0 (PMC12369096; doi:10.1186/s12985-025-02899-0)
Supplement: Supplementary file 1 — Supplementary Material 1 [file 12985_2025_2899_MOESM1_ESM.docx]

**SUPPLEMENTARY APPENDIX**

# Supplemental Methods 1. PRISMA 2020 Main Checklist

| **Topic** | **No.** | **Item** | **Location where item is reported** |
| --- | --- | --- | --- |
| **TITLE** |  |  |  |
| **Title** | 1 | Identify the report as a systematic review. | MS. Pg.1 |
| **ABSTRACT** |  |  |  |
| **Abstract** | 2 | See the PRISMA 2020 for Abstracts checklist | NA |
| **INTRODUCTION** |  |  |  |
| **Rationale** | 3 | Describe the rationale for the review in the context of existing knowledge. | MS.;Pg.4 |
| **Objectives** | 4 | Provide an explicit statement of the objective(s) or question(s) the review addresses. | MS.;Pg.4 |
| **METHODS** |  |  |  |
| **Eligibility criteria** | 5 | Specify the inclusion and exclusion criteria for the review and how studies were grouped for the syntheses. | MS, Pg.5 |
| **Information sources** | 6 | Specify all databases, registers, websites, organizations, reference lists and other sources searched or consulted to identify studies. Specify the date when each source was last searched or consulted. | MS, Pg.5 |
| **Search strategy** | 7 | Present the full search strategies for all databases, registers, and websites, including any filters and limits used. | MS, Pg.5-6; Sup. Methods 3 |
| **Selection process** | 8 | Specify the methods used to decide whether a study met the inclusion criteria of the review, including how many reviewers screened each record and each report retrieved, whether they worked independently, and if applicable, details of automation tools used in the process. | MS, Pg.6 |
| **Data collection process** | 9 | Specify the methods used to collect data from reports, including how many reviewers collected data from each report, whether they worked independently, any processes for obtaining or confirming data from study investigators, and if applicable, details of automation tools used in the process. | MS, Pg.6 |
| **Data items** | 10a | List and define all outcomes for which data were sought. Specify whether all results that were compatible with each outcome domain in each study were sought (e.g., for all measures, time points, analyses), and if not, the methods used to decide which results to collect. | MS, Pg.6; Sup. Methods 4 |
|  | 10b | List and define all other variables for which data were sought (e.g., participant and intervention characteristics, funding sources). Describe any assumptions made about any missing or unclear information. | MS, Pg.6 |
| **Study risk of bias assessment** | 11 | Specify the methods used to assess risk of bias in the included studies, including details of the tool(s) used, how many reviewers assessed each study and whether they worked independently, and if applicable, details of automation tools used in the process. | MS, Pg.7 |
| **Effect measures** | 12 | Specify for each outcome the effect measure(s) (e.g., risk ratio, mean difference) used in the synthesis or presentation of results. | MS, Pg.7 |
| **Synthesis methods** | 13a | Describe the processes used to decide which studies were eligible for each synthesis (e.g., tabulating the study intervention characteristics and comparing against the planned groups for each synthesis (item 5)). | MS, Pg.7 |
|  | 13b | Describe any methods required to prepare the data for presentation or synthesis, such as handling of missing summary statistics, or data conversions. | MS, Pg.7 |
|  | 13c | Describe any methods used to tabulate or visually display results of individual studies and syntheses. | MS, Pg.7 |
|  | 13d | Describe any methods used to synthesize results and provide a rationale for the choice(s). If meta-analysis was performed, describe the model(s), method(s) to identify the presence and extent of statistical heterogeneity, and software package(s) used. | MS, Pg.7 |
|  | 13e | Describe any methods used to explore possible causes of heterogeneity among study results (e.g., subgroup analysis, meta-regression). | MS, Pg.7 |
|  | 13f | Describe any sensitivity analyses conducted to assess robustness of the synthesized results. | MS, Pg.7 |
| **Reporting bias assessment** | 14 | Describe any methods used to assess risk of bias due to missing results in a synthesis (arising from reporting biases). | MS, Pg.7 |
| **Certainty assessment** | 15 | Describe any methods used to assess certainty (or confidence) in the body of evidence for an outcome. | NA |
| **RESULTS** |  |  |  |
| **Study selection** | 16a | Describe the results of the search and selection process, from the number of records identified in the search to the number of studies included in the review, ideally using a flow diagram. | Figure 1 |
|  | 16b | Cite studies that might appear to meet the inclusion criteria, but which were excluded, and explain why they were excluded. | NA |
| **Study characteristics** | 17 | Cite each included study and present its characteristics. | Table 1 |
| **Risk of bias in studies** | 18 | Present assessments of risk of bias for each included study. | MS; Pg.10 |
| **Results of individual studies** | 19 | For all outcomes, present, for each study: (a) summary statistics for each group (where appropriate) and (b) an effect estimates and its precision (e.g., confidence/credible interval), ideally using structured tables or plots. | MS; Pg.9-10; Fig.2-5 |
| **Results of syntheses** | 20a | For each synthesis, briefly summarize the characteristics and risk of bias among contributing studies. | MS; Pg.9-10 |
|  | 20b | Present results of all statistical syntheses conducted. If meta-analysis was done, present for each the summary estimate and its precision (e.g., confidence/credible interval) and measures of statistical heterogeneity. If comparing groups, describe the direction of the effect. | MS; Pg.9-10 |
|  | 20c | Present results of all investigations of possible causes of heterogeneity among study results. | MS; Pg.9-10 |
|  | 20d | Present results of all sensitivity analyses conducted to assess the robustness of the synthesized results. | MS; Pg.9-10 |
| **Reporting biases** | 21 | Present assessments of risk of bias due to missing results (arising from reporting biases) for each synthesis assessed. | MS; Pg.10 |
| **Certainty of evidence** | 22 | Present assessments of certainty (or confidence) in the body of evidence for each outcome assessed. | NA |
| **DISCUSSION** |  |  |  |
| **Discussion** | 23a | Provide a general interpretation of the results in the context of other evidence. | MS; Pg.12-15 |
|  | 23b | Discuss any limitations of the evidence included in the review. | MS; Pg.12-15 |
|  | 23c | Discuss any limitations of the review processes used. | MS; Pg.12-15 |
|  | 23d | Discuss implications of the results for practice, policy, and future research. | MS; Pg.12-15 |
| **OTHER INFORMATION** |  |  |  |
| **Registration and protocol** | 24a | Provide registration information for the review, including register name and registration number, or state that the review was not registered. | PROSPERO; CRD42024562860 |
|  | 24b | Indicate where the review protocol can be accessed, or state that a protocol was not prepared. | https://www.crd.york.ac.uk/PROSPERO/display_record.php?RecordID=562860 |
|  | 24c | Describe and explain any amendments to information provided at registration or in the protocol. | NA |
| **Support** | 25 | Describe sources of financial or non-financial support for the review, and the role of the funders or sponsors in the review. | MS; Pg.1 |
| **Competing interests** | 26 | Declare any competing interests of review authors. | MS; Pg.1 |
| **Availability of data, code and other materials** | 27 | Report which of the following are publicly available and where they can be found template data collection forms; data extracted from included studies; data used for all analyses; analytic code; any other materials used in the review. | NA |

^Abbreviations: MS, manuscript; sup., supplement.^

# Supplemental Methods 2. PRISMA Abstract Checklist

| **Topic** | **No.** | **Item** | **Reported?** |
| --- | --- | --- | --- |
| **TITLE** |  |  |  |
| **Title** | 1 | Identify the report as a systematic review. | Yes |
| **BACKGROUND** |  |  |  |
| **Objectives** | 2 | Provide an explicit statement of the main objective(s) or question(s) the review addresses. | Yes |
| **METHODS** |  |  |  |
| **Eligibility criteria** | 3 | Specify the inclusion and exclusion criteria for the review. | Yes |
| **Information sources** | 4 | Specify the information sources (e.g., databases, registers) used to identify studies and the date when each was last searched. | Yes |
| **Risk of bias** | 5 | Specify the methods used to assess risk of bias in the included studies. | No |
| **Synthesis of results** | 6 | Specify the methods used to present and synthesize results. | Yes |
| **RESULTS** |  |  |  |
| **Included studies** | 7 | Give the total number of included studies and participants and summarize relevant characteristics of studies. | Yes |
| **Synthesis of results** | 8 | Present results for main outcomes, preferably indicating the number of included studies and participants for each. If meta-analysis was done, report the summary estimate and confidence/credible interval. If comparing groups, indicate the direction of the effect (i.e., which group is favored). | Yes |
| **DISCUSSION** |  |  |  |
| **Limitations of evidence** | 9 | Provide a brief summary of the limitations of the evidence included in the review (e.g., study risk of bias, inconsistency and imprecision). | No |
| **Interpretation** | 10 | Provide a general interpretation of the results and important implications. | No |
| **OTHER** |  |  |  |
| **Funding** | 11 | Specify the primary source of funding for the review. | No |
| **Registration** | 12 | Provide the register name and registration number. | Yes |

# Supplemental Methods 3. Details of the Search Strategy

| **Search Strategy for each database** | |
| --- | --- |
| **Pubmed** | (“COVID-19”[Mesh] OR “SARS-CoV-2” OR Coronavirus OR “COVID19” OR “SARS-CoV-2”[Mesh] OR COVID19 OR SARSCOV2) AND (“mild to moderate” OR “mild” OR “moderate”) AND (nirmatrelvir OR “ensitrelvir” [Supplementary Concept] OR “SIM0417” OR “nirmatrelvir and ritonavir drug combination” [Supplementary Concept] OR “nirmatrelvir, ritonavir drug combination” OR “nirmatrelvir, ritonavir” OR “PF-07321332” OR “Paxlovid” OR “simnotrelvir” OR “Ritonavir”[Mesh] OR “GST-HG171”) AND (“randomized controlled trial”[pt] OR “controlled clinical trial”[pt] OR randomized[tiab] OR placebo[tiab] OR “drug therapy”[sh] OR randomly[tiab] OR trial[tiab] OR groups[tiab]) |
| **EMBASE** | (COVID-19/exp OR SARS-CoV-2 OR Coronavirus OR COVID19 OR SARS-CoV-2/exp OR COVID19 OR SARSCOV2) AND (‘mild to moderate’ OR mild OR moderate) AND (ensitrelvir:tn OR SIM0417 OR ‘nirmatrelvir and ritonavir drug combination’:tn OR ‘nirmatrelvir, ritonavir drug combination’ OR ‘nirmatrelvir, ritonavir’ OR PF-07321332 OR Paxlovid OR simnotrelvir OR Ritonavir/exp OR GST-HG171) AND (term:it OR term:it OR randomized:ti,ab OR placebo:ti,ab OR “Drug Therapy” OR randomly:ti,ab OR trial:ti,ab OR groups:ti,ab) |
| **Cochrane Library** | (“COVID-19” OR “SARS-CoV-2” OR Coronavirus OR COVID19 OR COVID19 OR SARSCOV2) AND ("mild to moderate" OR mild OR moderate) AND ( ensitrelvir OR SIM0417 OR nirmatrelvir OR ”nirmatrelvir and ritonavir drug combination” OR "nirmatrelvir, ritonavir drug combination" OR "nirmatrelvir, ritonavir" OR PF-07321332 OR Paxlovid OR simnotrelvir OR “Ritonavir” OR GST-HG171) (“COVID-19” OR “SARS-CoV-2” OR Coronavirus OR COVID19 OR COVID19 OR SARSCOV2) AND ("mild to moderate" OR mild OR moderate) AND ( ensitrelvir OR SIM0417 OR nirmatrelvir OR ”nirmatrelvir and ritonavir drug combination” OR "nirmatrelvir, ritonavir drug combination" OR "nirmatrelvir, ritonavir" OR PF-07321332 OR Paxlovid OR simnotrelvir OR “Ritonavir” OR GST-HG171) |

**Supplemental Methods 4. Reconstructed KM Curves Data Extraction**

To collect the individual participant data (IPD), we followed Guyot et al.[1] method of reconstructing the IPD through the Kaplan-Meier Curve reports in some of the included studies. After downloading and digitizing the images of the Kaplan-Meier curves, the step function values and step timing were obtained, and the time-to-event data was calibrated using the total number of patients and number-at-risk tables. Finally, the individual patient survival data was obtained using the inverted Kaplan-Meier product limit equations, and the survival data was calculated using a Cox regression model.

**Supplemental Methods 5. Definitions of Outcomes**

| **Outcome** | **Definition** |
| --- | --- |
| **RNA Viral Load** | Quantity of RNA copies per milliliter of the viral transport medium or per swab, or as the test-specific cycle threshold (Ct) value. [2] |
| **Adverse events** | A negative alteration in the health of a participant, including abnormal laboratory results that are not necessarily related to the intervention/treatment of the study. [3] |
| **Serious/Severe adverse events** | An adverse event that leads to death, birth anomalies, results in a persistent or significant incapacity/disability, is life-threatening, requires inpatient hospitalization or prolongation of an existing hospital stay, necessitates medical or surgical intervention to prevent one of the other outcomes. [4] |
| **Median recovery time** | Median recovery time was defined as the number of days from the initiation of treatment to the point when a patient first met the criteria for clinical recovery, as defined in each study. [5-14] |

**Supplemental Methods 6. Post-hoc protocol amendments**

The initial protocol specified inclusion of hospitalized patients only, however, during the review process, we expanded the eligibility to include both hospitalized and non-hospitalized patients with mild to moderate COVID-19. In addition, we did a post-hoc subgroup analysis for different 3CL protease inhibitor types.

# Supplemental Results 1. Eligibility and Recovery/Alleviation Criteria

| **Study (Year)** | **Inclusion Criteria** | **Exclusion Criteria** | **Definition of Recovery/Alleviation** |
| --- | --- | --- | --- |
| Bei Wang et al. (2024) | Adults 18–70, confirmed SARS-CoV-2 ≤5 days, Ct ≤30, mild/moderate COVID-19 | Severe/critical cases, BMI ≥30, antiviral use, systemic infections, low SpO₂ (≤93%) | All symptoms return to normal and remain so ≥2 days; continuous recovery = time to first such point |
| Bin Cao et al. (2024) | Adults ≥18, COVID-19 symptom onset ≤3 days, mild/moderate illness | Need for ventilation, serious comorbidities, CYP3A4 interactions | All 11 symptoms score 0 for 2 days (recovery); ≤1 (alleviation) |
| Fuxiang Wang et al. (2023) | Adults 18–75, symptom onset ≤3 days, positive test ≤5 days | Severe disease, SpO₂ ≤93%, liver/renal issues, HIV, drug use | All 9 symptoms score 0 or 1 for ≥1 day |
| Jiang et al. (2024) | Mild/moderate COVID-19, ≥2 symptoms in 72h, ≥1 symptom score ≥2 | Severe/critical COVID-19, ventilation expected, asthma/COPD flare | Sustained recovery of 11/5 symptoms for ≥2 days |
| Hammond et al. (2024) | Adults ≥18, symptoms ≤5 days, vaccinated if at risk | Excluded patients with risk factors (per protocol) | All symptoms reduced to mild/none for 4 consecutive days |
| Hongzhou Lu et al. (2024) | Adults ≥18, RT-PCR+ ≤5 days, ≥2 symptoms in 72h | Severe/critical cases, recent vax/treatment, drug interactions | All 11 symptoms score 0 (recovery) or ≤1 (relief) for 2 days |
| Mukae et al. (2022, 1) | Age 12–69, symptoms ≤120h, ≥1 moderate/severe symptom | SpO₂ ≤93%, oxygen required | Symptoms improve to mild/none and persist ≥24h |
| Mukae et al. (2022, 2) | Age 12–69, test+ ≤120h, ≥1 moderate/severe symptom | SpO₂ ≤93%, systemic infections, liver/kidney disease, drugs, pregnancy | Same as Mukae (1) |
| Yangqing Zhan et al. (2024) | Adults 18–75, symptoms ≤48h, NAT+ ≤120h, mild/moderate | Severe disease, hospitalization, liver/kidney dysfunction | All 9 symptoms score 0 or 1 for ≥1 day |
| Yotsuyanagi et al. (2024) | Age 12–69, symptoms ≤120h, ≥1 moderate/severe symptom | SpO₂ ≤93%, exacerbation, liver/kidney disease | Resolution of 5 symptoms for ≥24h; improved from baseline |

## **Supplemental Results 2. Evaluated COVID-19 symptoms per study**

| **Symptom** | **Mukae 2022 (1) et al.** | **Mukae 2022 (2)** | **Bin Cao 2024 et al.** | **Jiang 2024 et al.** | **Hammond 2024 et al.** | **Honghzou Lu 2024** | **Yangqing Zhan 2024** | **Bei Wang 2023 et al.** | **Yotsunagi 2024** | **Fuxiang Wang 2023** |
| --- | --- | --- | --- | --- | --- | --- | --- | --- | --- | --- |
| **Stuffy or runny nose** | X | X | X | X | X | X | X | X | X | X |
| **Sore throat** | X | X | X | X | X | X | X | X | X | X |
| **Shortness of breath** | X | X | X | X | X | X | X | X | X | X |
| **Cough** | X | X | X | X | X | X | X | X | X | X |
| **Low energy or tiredness** | X | X |  |  |  | X |  | X | X | X |
| **Muscle or body aches** | X | X | X | X | X | X | X | X | X | X |
| **Headache** | X | X | X | X | X | X | X | X | X | X |
| **Chills or shivering** | X | X | X | X | X | X | X | X | X | X |
| **Feeling hot or feverish** | X | X | X | X | X | X | X | X | X | X |
| **Nausea** | X | X | X | X | X | X | X | X | X |  |
| **Vomiting** | X | X | X | X | X | X | X | X | X |  |
| **Diarrhea** | X | X | X | X | X | X | X | X | X |  |
| **Sense of smell in the last 24 hours** |  |  |  |  |  | X |  | X | X |  |
| **Sense of taste in the last 24 hours** |  |  |  |  |  | X |  | X | X |  |

## **Supplemental Results 3. Reconstructed KM Curves**

In this section, we will display our reconstructed Kaplan Meier Curve using the Guyot et al. method for obtaining individual patient data as reported in Supplementary Methods 5

**Cao et al. 2024**


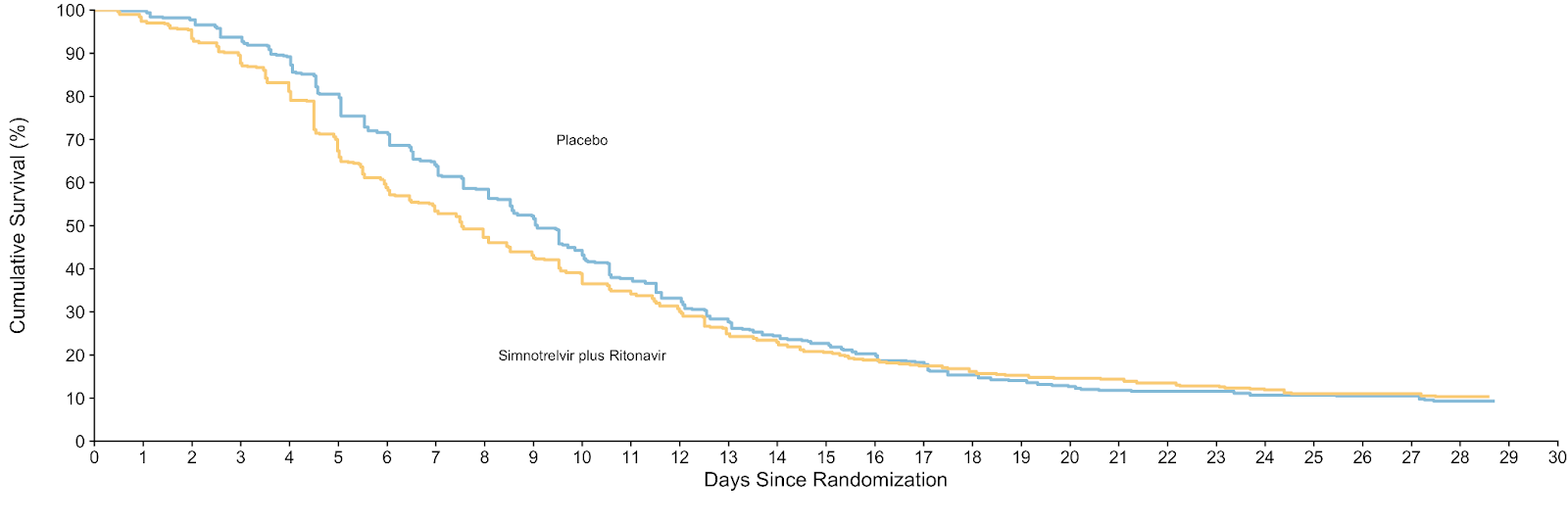


**Yotsuyanagi et al. 2024**

**125mg                                        250mg**


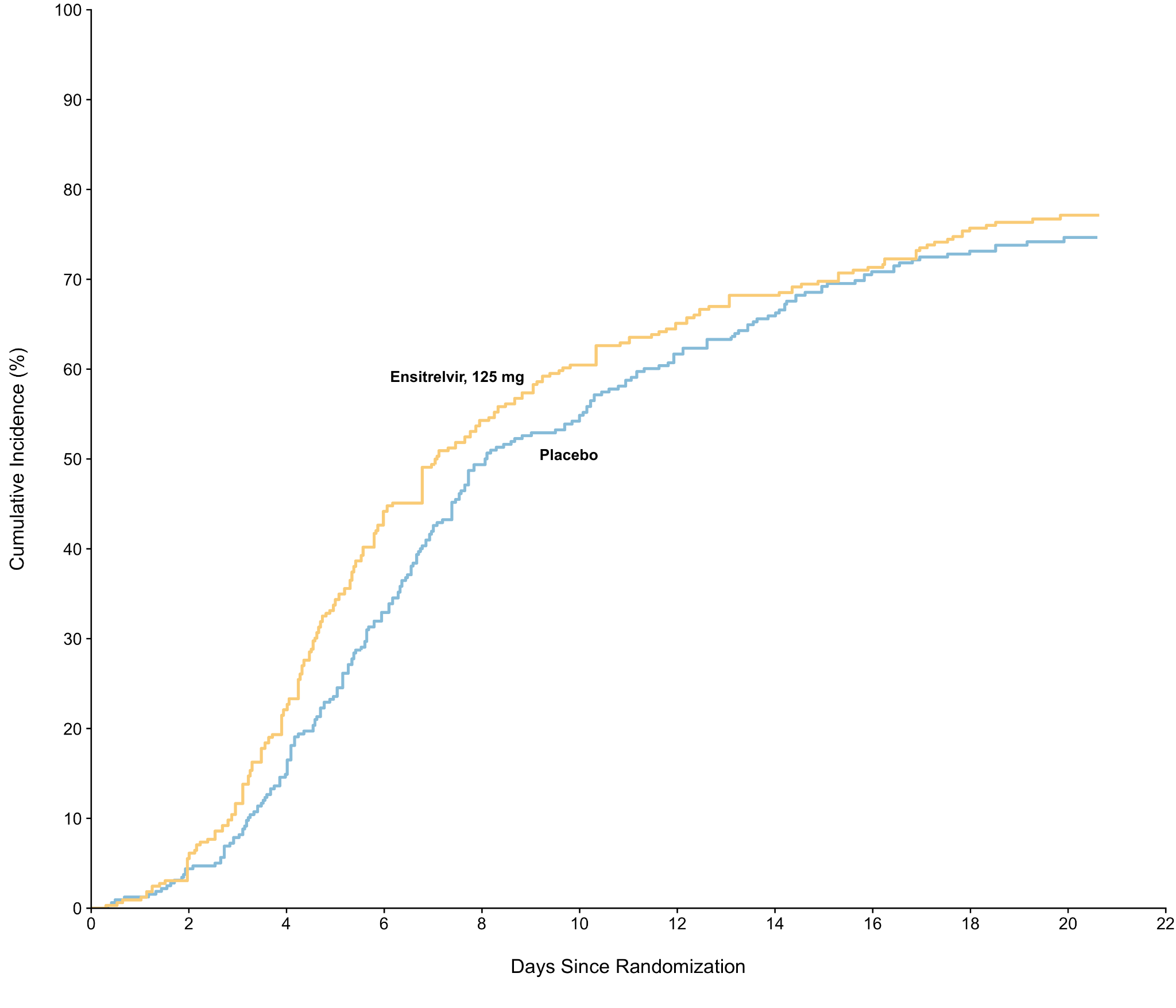

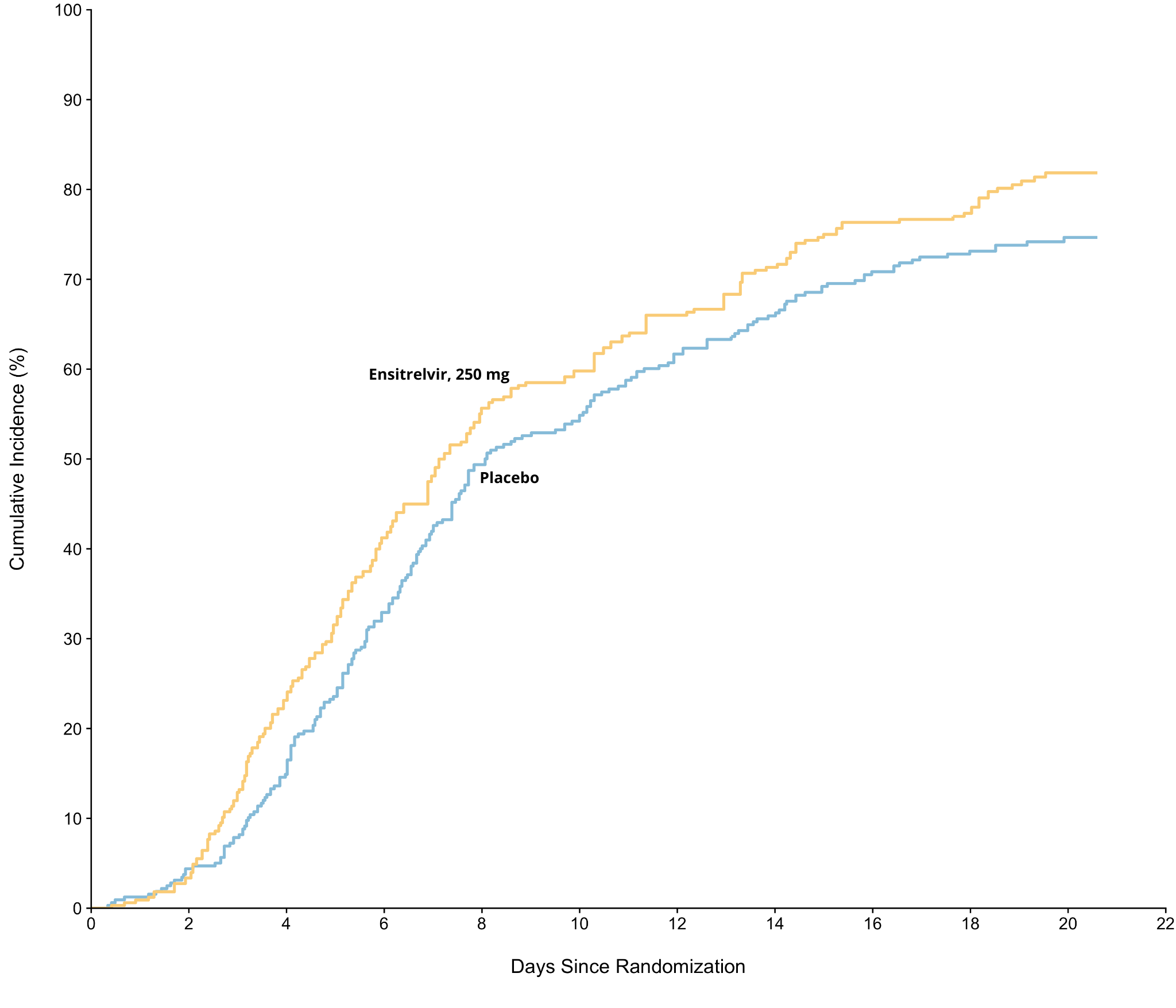


**Supplemental Figure 1 Any Adverse Events**


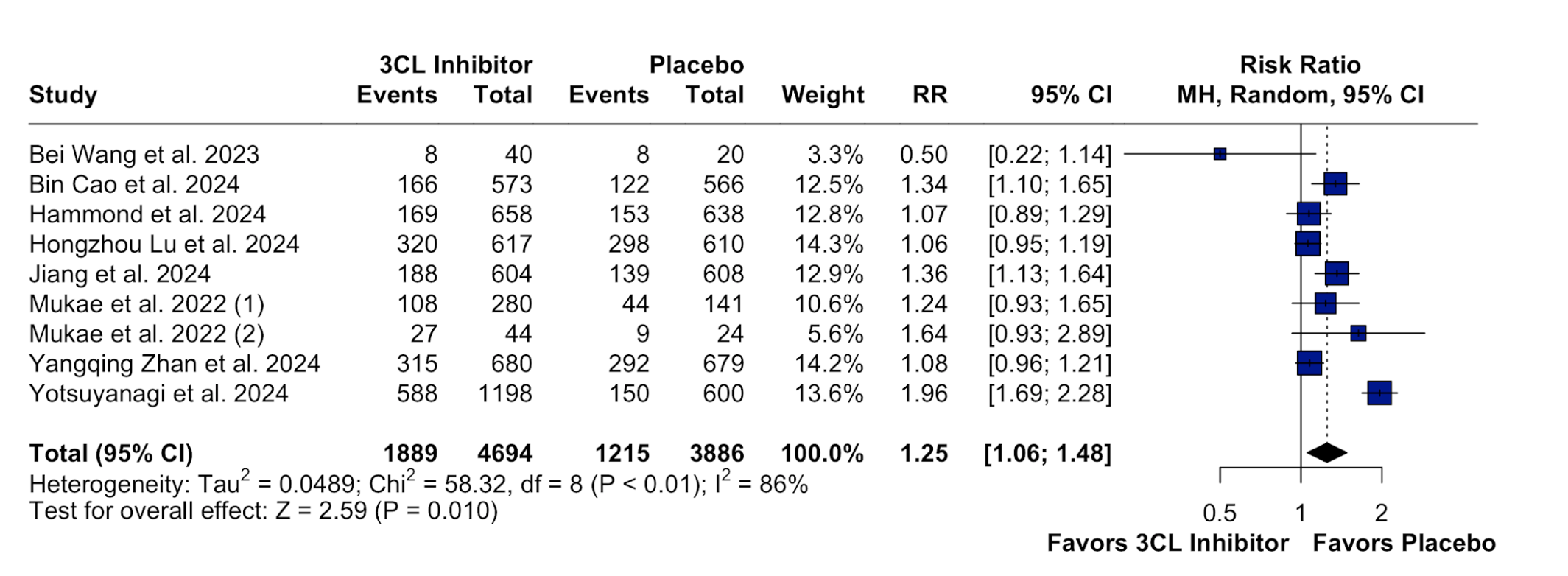


*Legend:* There is a higher risk of any adverse events of COVID-19 in patients in 3CL inhibitors therapy compared to placebo. *Abbreviations:* 3CL:3-chymotrypsin-like; CI: Confidence Interval; IV: inverse variance; MH: Mantel-Haenszel; RR: Risk Ratio

**Supplemental Figure 2 – Viral Load**

**Supplemental Figure 2A: Viral Load in 48 hours**


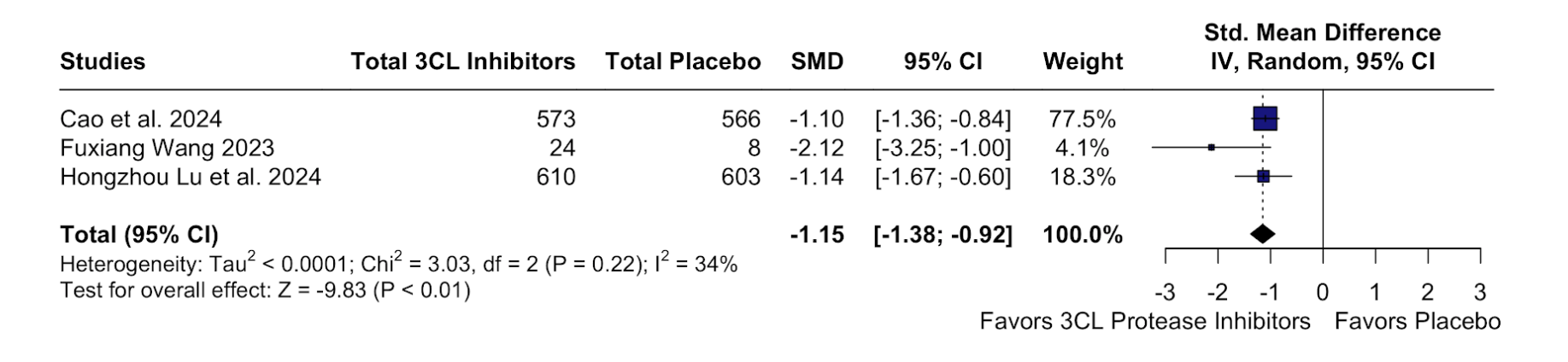


**Supplemental Figure 2B: Viral Load in 72 hours**


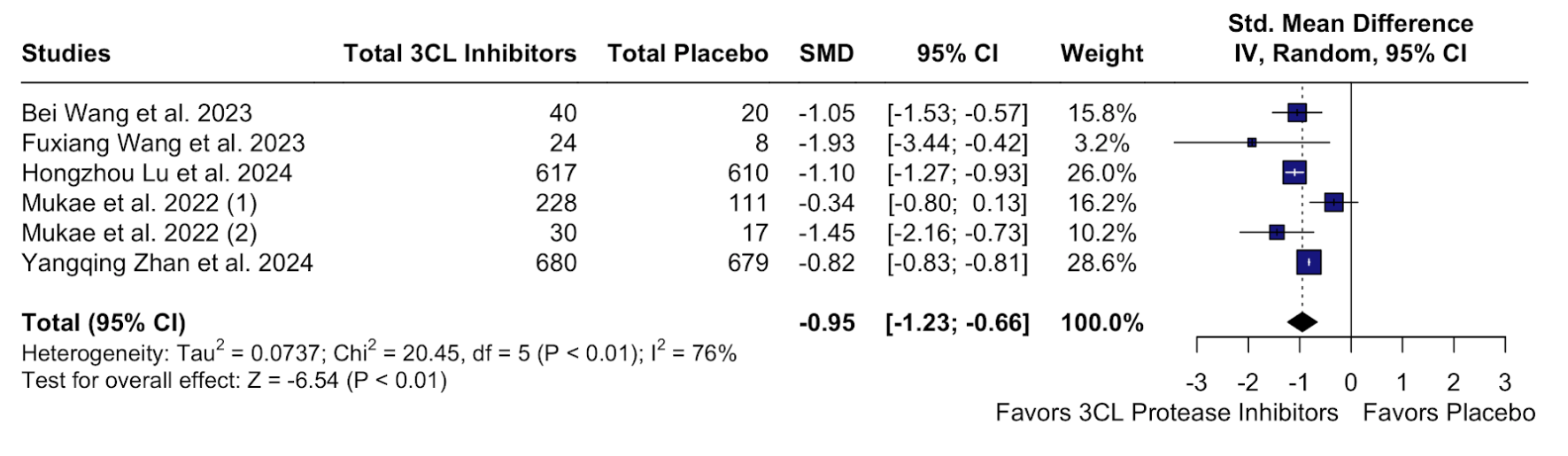


**Supplemental Figure 2C: Viral Load in 96 hours**


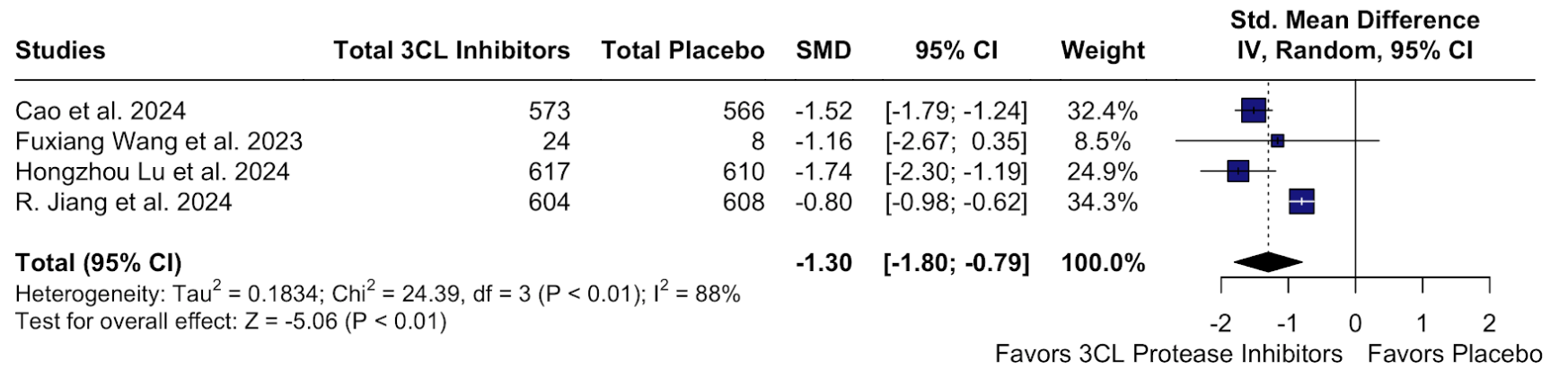


**Supplemental Figure 2D: Viral Load in 120 hours**

**
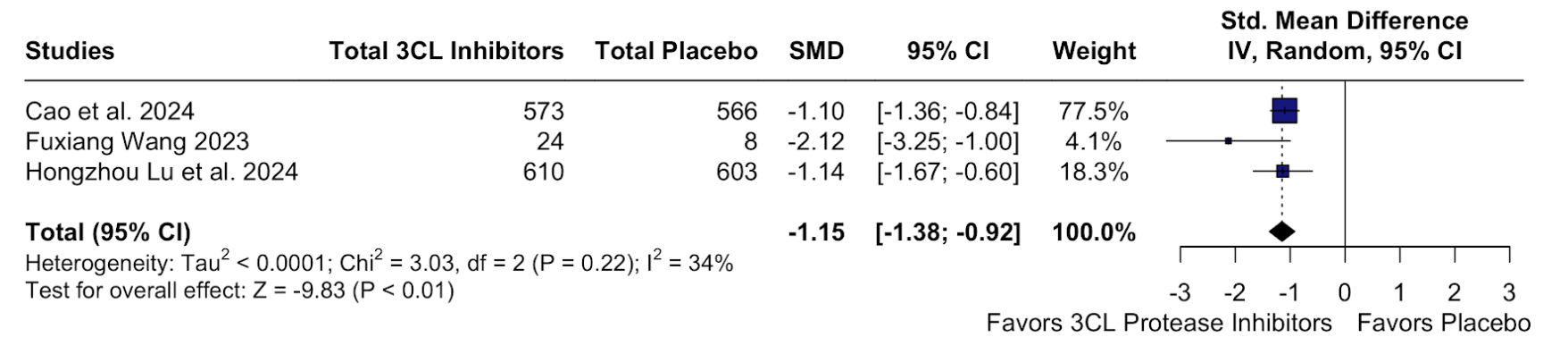
**

*Legend:* There is a lower viral load of COVID-19 in patients in 3CL inhibitors therapy compared to placebo. *Abbreviations:* 3CL:3-chymotrypsin-like; CI: Confidence Interval; IV: inverse variance; MH: Mantel-Haenszel; RR: Risk Ratio

**Supplemental Figure 3: Subgroup Analysis Viral Load in 72 hours Ensitrelvir 125 mg versus Ensitrelvir 250 mg**


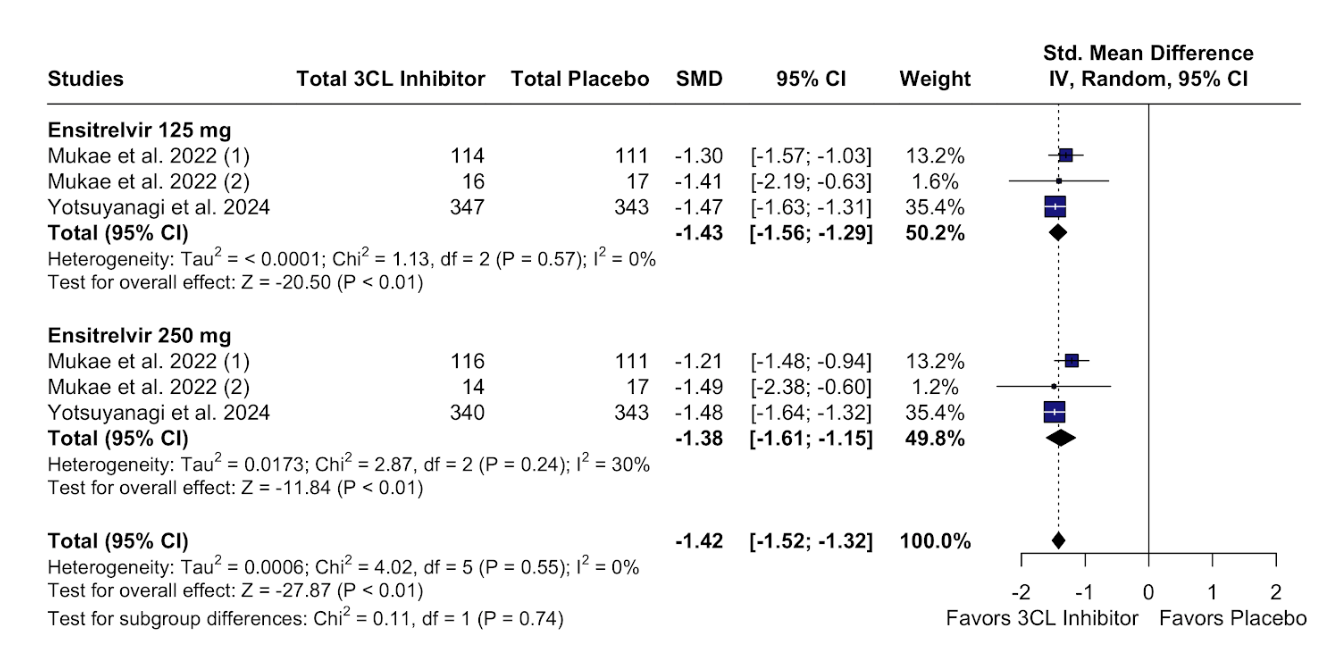


*Legend:* There is a higher viral load of COVID-19 in patients in 3CL inhibitors therapy compared to placebo. *Abbreviations:* 3CL:3-chymotrypsin-like; CI: Confidence Interval; IV: inverse variance; MH: Mantel-Haenszel; RR: Risk Ratio

**Supplemental Figure 4: Post-hoc Subgroup Analysis for different 3CL inhibitor drugs**


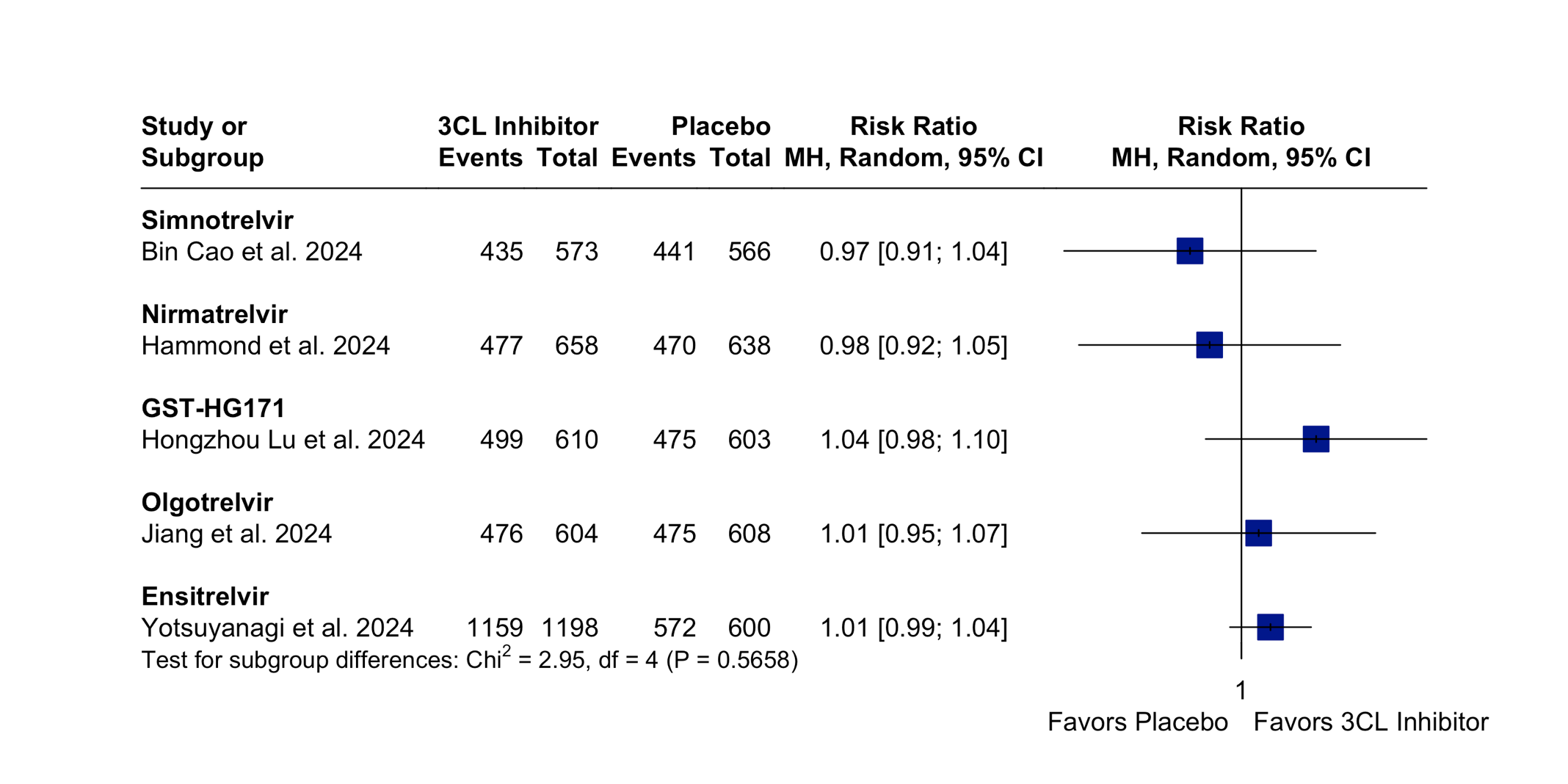


**Supplemental Figure 5. Trial Sequential Analysis(TSA)**

**Supplemental Figure 5A: TSA for number of resolutions**

**
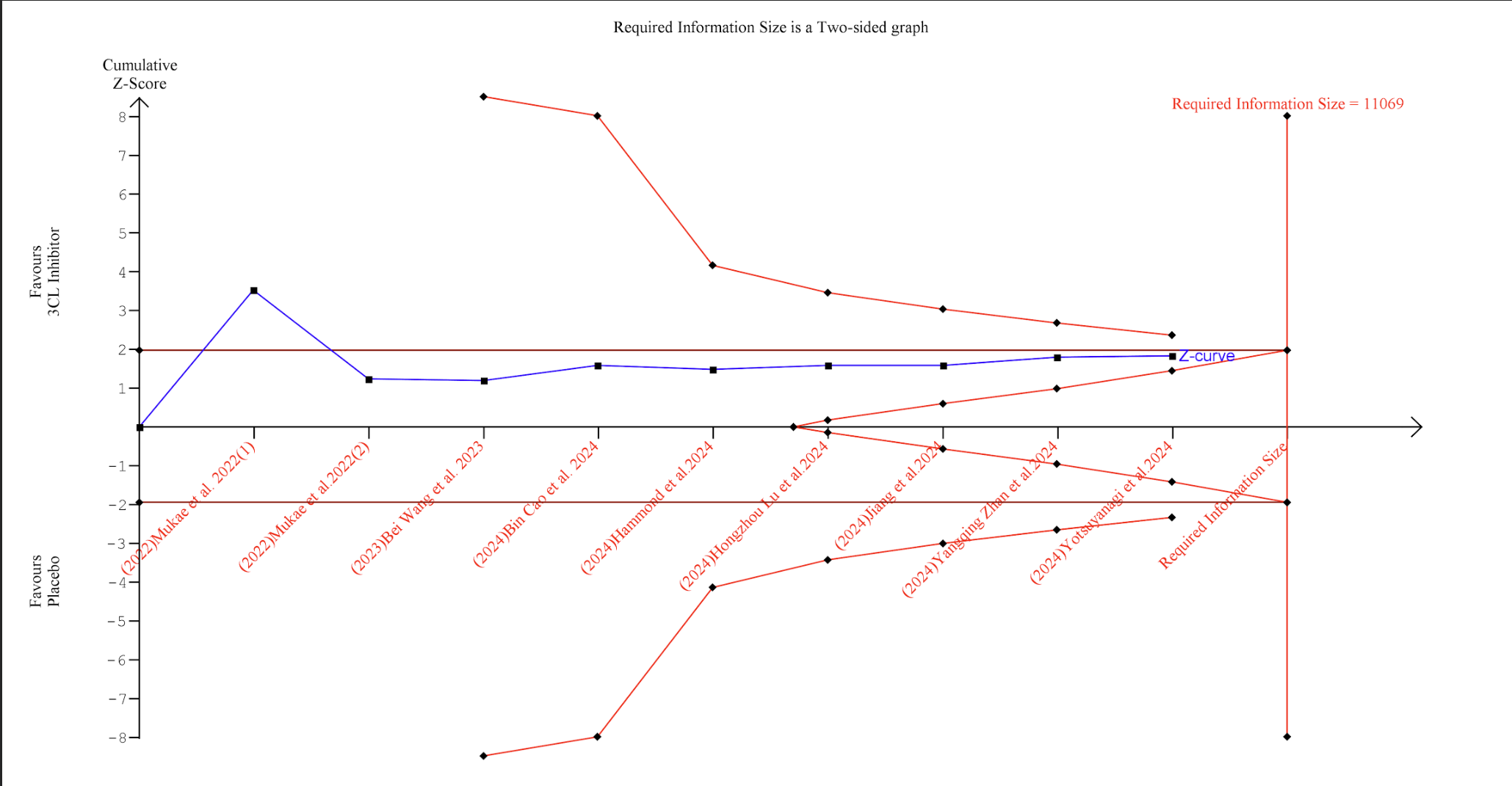
**

**Supplemental Figure 5B: TSA for Serious or Severe Adverse Events**

**
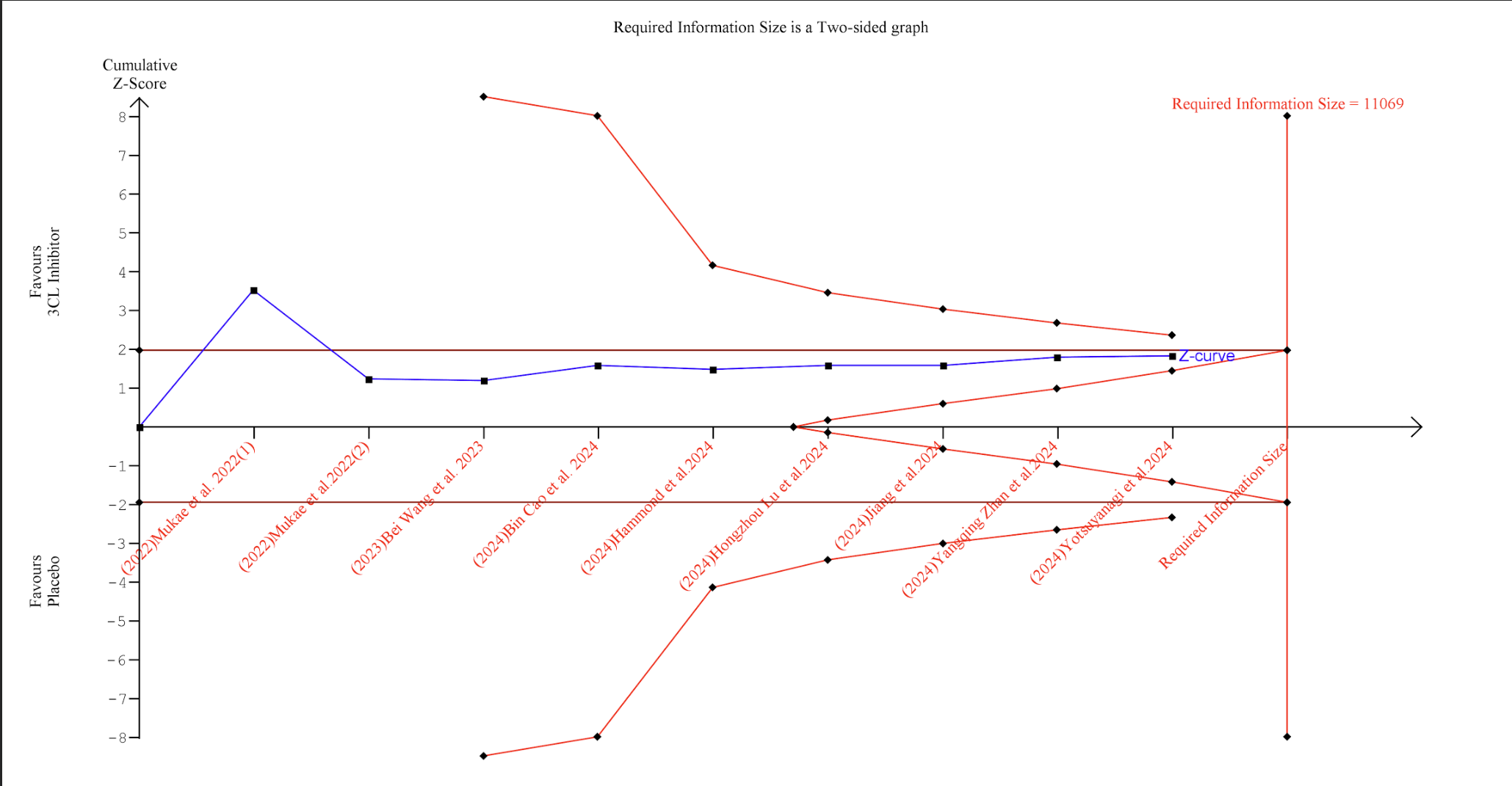
**

**Supplemental Figure 6. Risk of Bias 2 of All Included Studies**

**Supplemental Figure 6A:** "Traffic light" plot of the domain-level judgments for each study


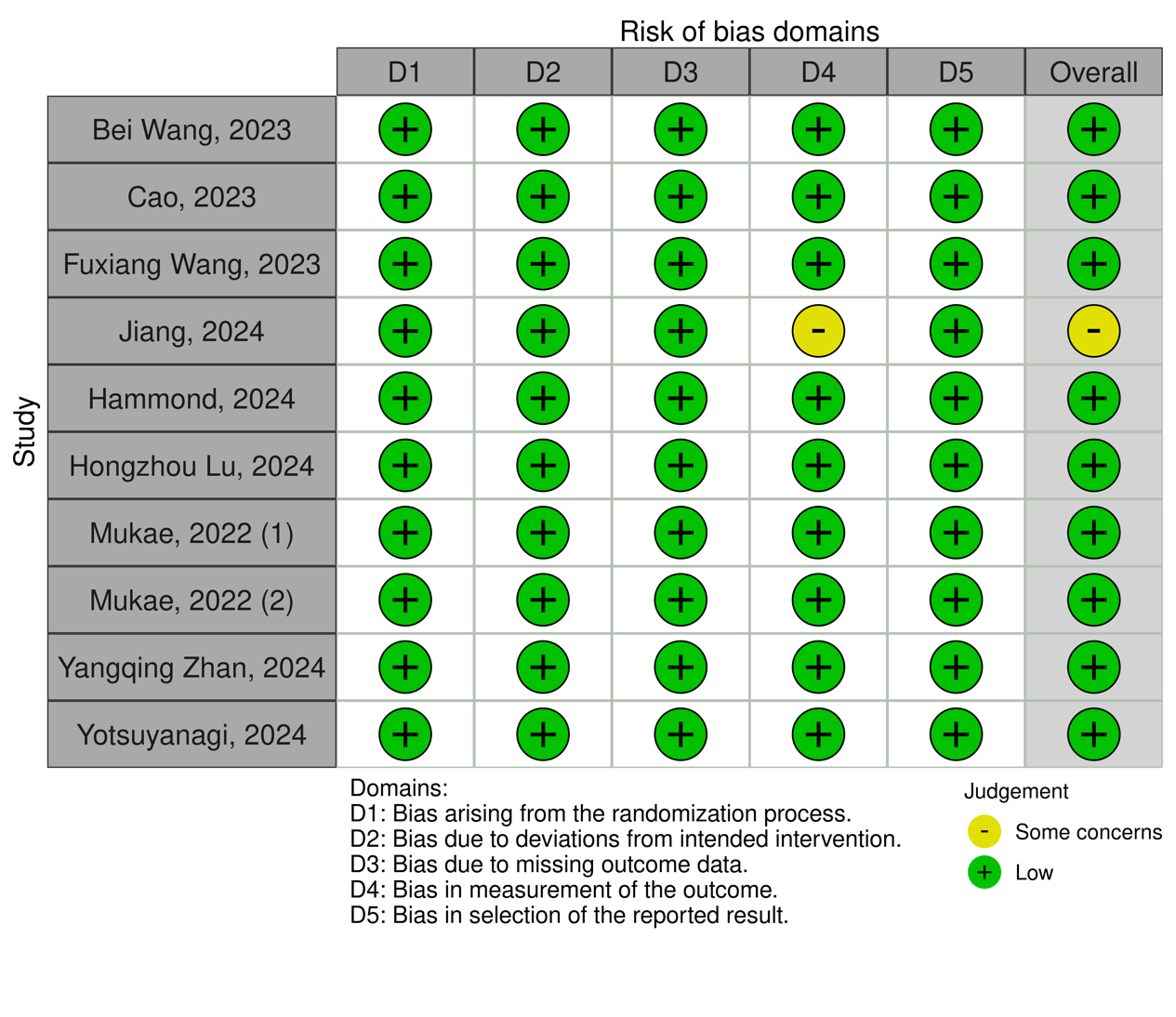


**Supplemental Figure 6B:** Summary of overall weighted bar plot of risk-of-bias judgments within each bias domain.

**
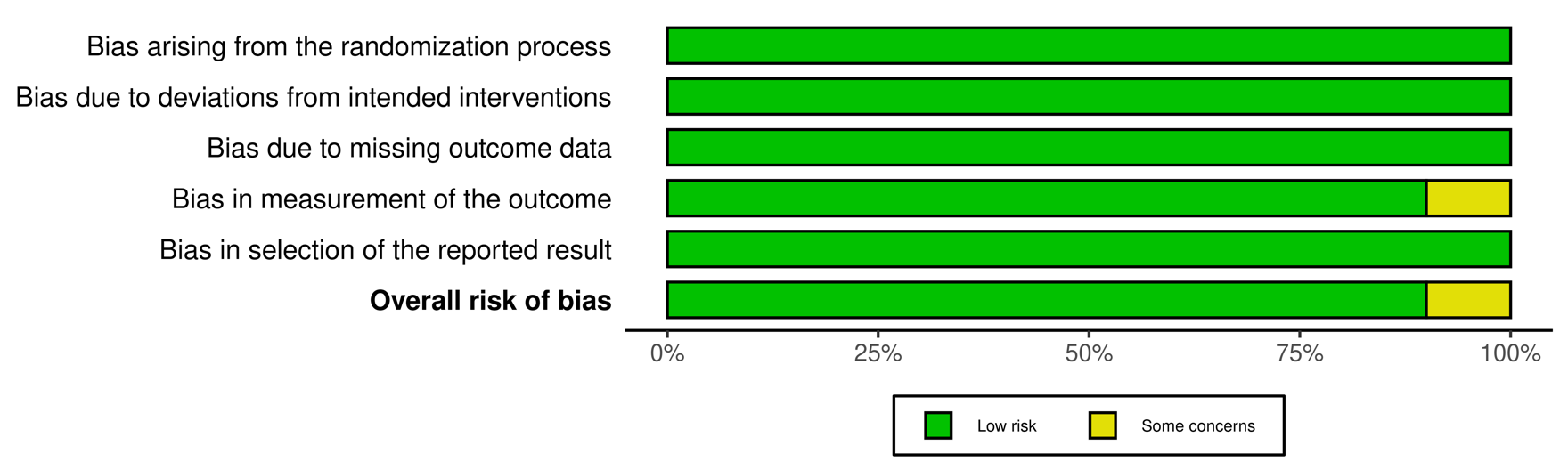
**

**Supplemental Figure 6C: Funnel Plot for the primary endpoint**

**
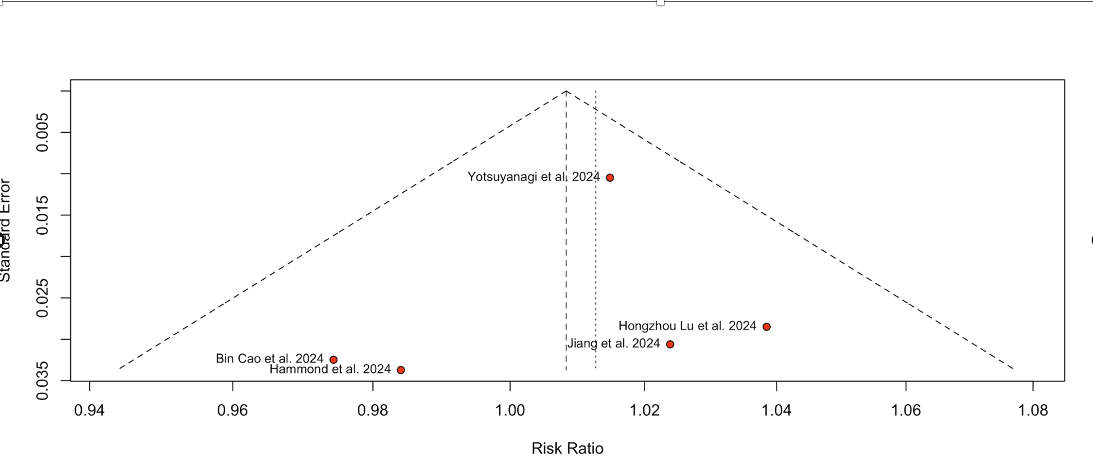
**

*Legend:* There is no asymmetry in the funnel plot suggesting no small study effect.

**Supplementary References**

[1] Guyot P, Ades A, Ouwens MJ, Welton NJ. Enhanced secondary analysis of survival data: reconstructing the data from published Kaplan-Meier survival curves. BMC Med Res Methodol 2012;12:9. https://doi.org/10.1186/1471-2288-12-9.

[2] Puhach O, Meyer B, Eckerle I. SARS-CoV-2 viral load and shedding kinetics. Nat Rev Microbiol 2022. https://doi.org/10.1038/s41579-022-00822-w.

[3] ClinicalTrials.gov. Study Basics: Glossary [Internet]. Available from: https://clinicaltrials.gov/study-basics/glossary. Accessed: 2024 Aug 6. n.d.

[4] https://www.fda.gov/safety/reporting-serious-problems-fda/what-serious-adverse-event n.d.

[5] Wang B, Li H, Cai M, Lin Z, Ou X, Wu S, et al. Antiviral efficacy of RAY1216 monotherapy and combination therapy with ritonavir in patients with COVID-19: a phase 2, single centre, randomised, double-blind, placebo-controlled trial. EClinicalMedicine 2023;63:102189. https://doi.org/10.1016/j.eclinm.2023.102189.

[6] Cao B, Wang Y, Lu H, Huang C, Yang Y, Shang L, et al. Oral Simnotrelvir for Adult Patients with Mild-to-Moderate Covid-19. N Engl J Med 2024;390:230–41. https://doi.org/10.1056/NEJMoa2301425.

[7] Wang F, Xiao W, Tang Y, Cao M, Shu D, Asakawa T, et al. Efficacy and safety of SIM0417 (SSD8432) plus ritonavir for COVID-19 treatment: a randomised, double-blind, placebo-controlled, phase 1b trial. Lancet Reg Health West Pac 2023;38:100835. https://doi.org/10.1016/j.lanwpc.2023.100835.

[8] Jiang R, Han B, Xu W, Zhang X, Peng C, Dang Q, et al. Olgotrelvir as a Single-Agent Treatment of Nonhospitalized Patients with Covid-19. NEJM Evidence 2024;3:EVIDoa2400026. https://doi.org/10.1056/EVIDoa2400026.

[9] Hammond J, Leister-Tebbe H, Gardner A, Abreu P, Bao W, Wisemandle W, et al. Oral Nirmatrelvir for High-Risk, Nonhospitalized Adults with Covid-19. N Engl J Med 2022;386:1397–408. https://doi.org/10.1056/NEJMoa2118542.

[10] Lu H, Zhang G, Mao J, Chen X, Zhan Y, Lin L, et al. Efficacy and safety of GST-HG171 in adult patients with mild to moderate COVID-19: a randomised, double-blind, placebo-controlled phase 2/3 trial. EClinicalMedicine 2024;71:102582. https://doi.org/10.1016/j.eclinm.2024.102582.

[11] Mukae H, Yotsuyanagi H, Ohmagari N, Doi Y, Sakaguchi H, Sonoyama T, et al. Efficacy and Safety of Ensitrelvir in Patients With Mild-to-Moderate Coronavirus Disease 2019: The Phase 2b Part of a Randomized, Placebo-Controlled, Phase 2/3 Study. Clin Infect Dis 2023;76:1403–11. https://doi.org/10.1093/cid/ciac933.

[12] Mukae H, Yotsuyanagi H, Ohmagari N, Doi Y, Imamura T, Sonoyama T, et al. A Randomized Phase 2/3 Study of Ensitrelvir, a Novel Oral SARS-CoV-2 3C-Like Protease Inhibitor, in Japanese Patients with Mild-to-Moderate COVID-19 or Asymptomatic SARS-CoV-2 Infection: Results of the Phase 2a Part. Antimicrob Agents Chemother 2022;66. https://doi.org/10.1128/aac.00697-22.

[13] Zhan Y, Lin Z, Liang J, Sun R, Li Y, Lin B, et al. Leritrelvir for the treatment of mild or moderate COVID-19 without co-administered ritonavir: a multicentre randomised, double-blind, placebo-controlled phase 3 trial. EClinicalMedicine 2024;67:102359. https://doi.org/10.1016/j.eclinm.2023.102359.

[14] Yotsuyanagi H, Ohmagari N, Doi Y, Yamato M, Bac NH, Cha BK, et al. Efficacy and Safety of 5-Day Oral Ensitrelvir for Patients With Mild to Moderate COVID-19: The SCORPIO-SR Randomized Clinical Trial. JAMA Netw Open 2024;7:e2354991. https://doi.org/10.1001/jamanetworkopen.2023.54991.
